# Irreducible Lateral Proximal Interphalangeal Joint Dislocation of the Fifth Toe in a Two-Year-Old Child: A Case Report With a Six-Year Follow-Up

**DOI:** 10.7759/cureus.106448

**Published:** 2026-04-04

**Authors:** Suraj Sajeev

**Affiliations:** 1 Department of Orthopedics and Traumatology, Kozhikode Medical College, Kozhikode, IND

**Keywords:** fifth toe, irreducible dislocation, open reduction, pediatric toe dislocation, proximal interphalangeal joint

## Abstract

Proximal interphalangeal (PIP) joint dislocations of the lesser toes are uncommon injuries, particularly in the pediatric population. Irreducible dislocations, where closed reduction fails due to interposed soft tissue, present a unique diagnostic and therapeutic challenge. We report a rare case of an irreducible lateral PIP joint dislocation of the fifth toe in a two-year-old child with a six-year follow-up. The child presented with blunt trauma to the right foot resulting from an abduction force to the fourth and fifth toes. Radiographs revealed a lateral dislocation of the right fifth toe PIP joint with an associated valgus angulated fracture of the proximal phalanx. Closed reduction failed in the emergency department. Open reduction under general anesthesia via a dorsal midline incision revealed the medial collateral ligament avulsed and interposed within the joint space, acting as a mechanical block. The ligament was removed, the joint reduced, and transarticular Kirschner wire (K-wire) fixation (1.6 mm) was performed. The K-wire was removed at three weeks. At six-year follow-up, clinical evaluation revealed no angular deformity, shortening, or scar tethering, with full range of motion and participation in age-appropriate activities. This case highlights that prompt open reduction, removal of interposed soft tissue, and temporary K-wire fixation can yield excellent long-term functional and cosmetic outcomes. Early recognition and timely surgery are essential in pediatric irreducible PIP joint dislocations to prevent chronic deformity.

## Introduction

Interphalangeal joint dislocations of the lesser toes are uncommon injuries that may result in chronic deformity and functional impairment if inadequately treated. The proximal interphalangeal (PIP) joint is most frequently involved, with the fifth toe particularly vulnerable due to its lateral position and susceptibility to abduction forces [1,2].

Most toe dislocations are reducible by closed means; however, irreducible dislocations occur when soft tissue structures such as the collateral ligaments, volar plate, or capsule become interposed within the joint space, preventing anatomic reduction [2-5]. These injuries require open surgical reduction to restore alignment and stability [6].

In the pediatric population, irreducible PIP joint dislocations are exceedingly rare and present additional diagnostic challenges [6-8]. Long-term outcomes in very young children remain sparsely reported. We describe the successful surgical management of an irreducible lateral PIP joint dislocation of the fifth toe in a two-year-old child, with a documented six-year follow-up.

## Case presentation

A two-year-old boy presented with acute right foot trauma following an abduction force applied to the fourth and fifth toes. Written informed consent was obtained from the patient’s parents for publication of this report and accompanying images, in accordance with the Declaration of Helsinki.

Examination revealed lateral deformity of the right fifth toe with swelling and tenderness at the PIP joint. Closed reduction attempts in the emergency department were unsuccessful. Neurovascular examination was normal.

**Figure 1:**
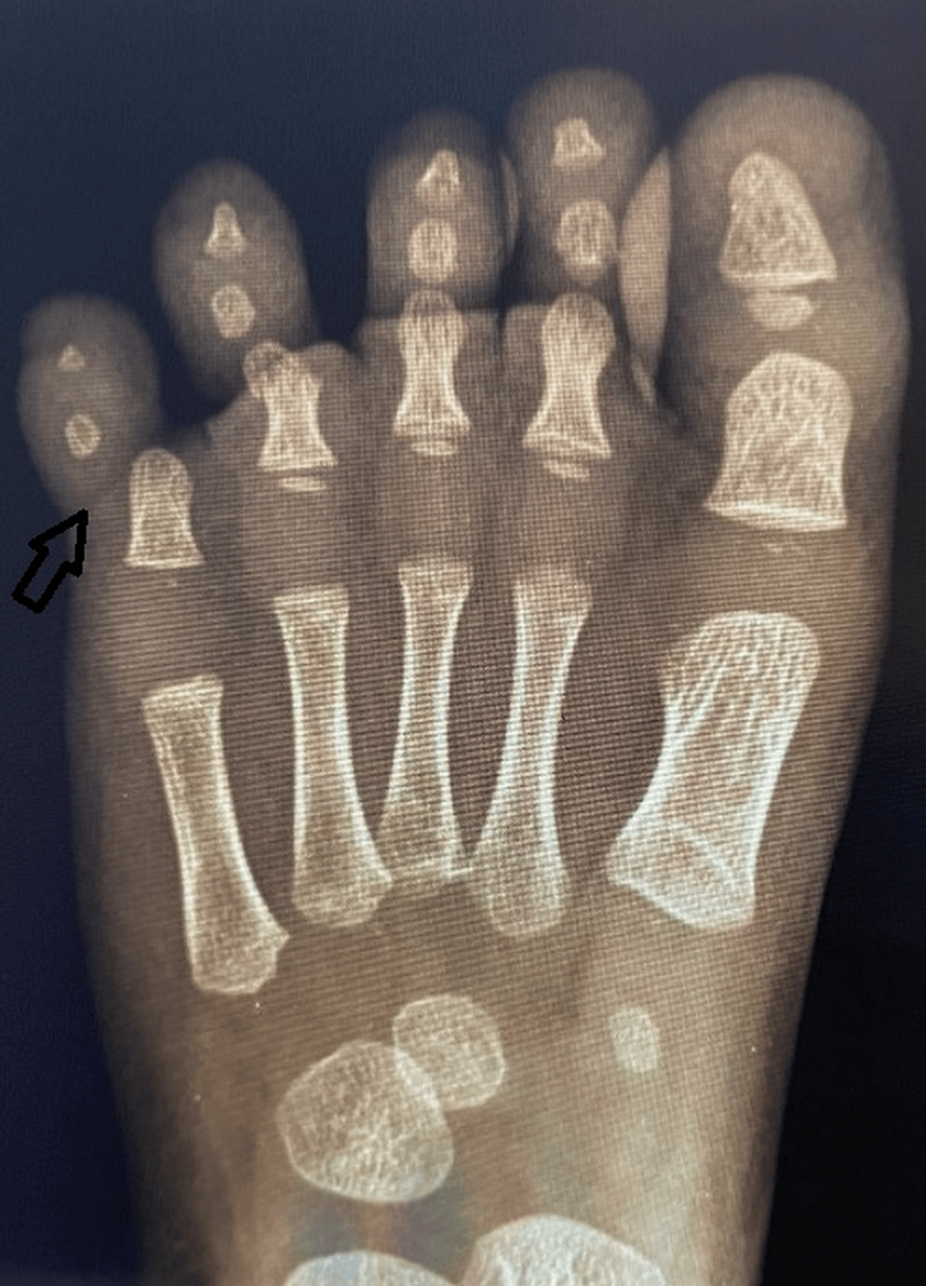
Preoperative anteroposterior radiograph of the right foot showing lateral dislocation of the PIP joint of the fifth toe (arrow) with associated valgus angulated fracture of the proximal phalanx PIP: proximal interphalangeal

Anteroposterior and oblique radiographs demonstrated a lateral dislocation of the fifth toe PIP joint with a mildly valgus angulated fracture of the proximal phalanx (Figure 1). The fourth toe injury was stable and treated conservatively. Given failed closed reduction and instability, open reduction was indicated.

**Figure 2:**
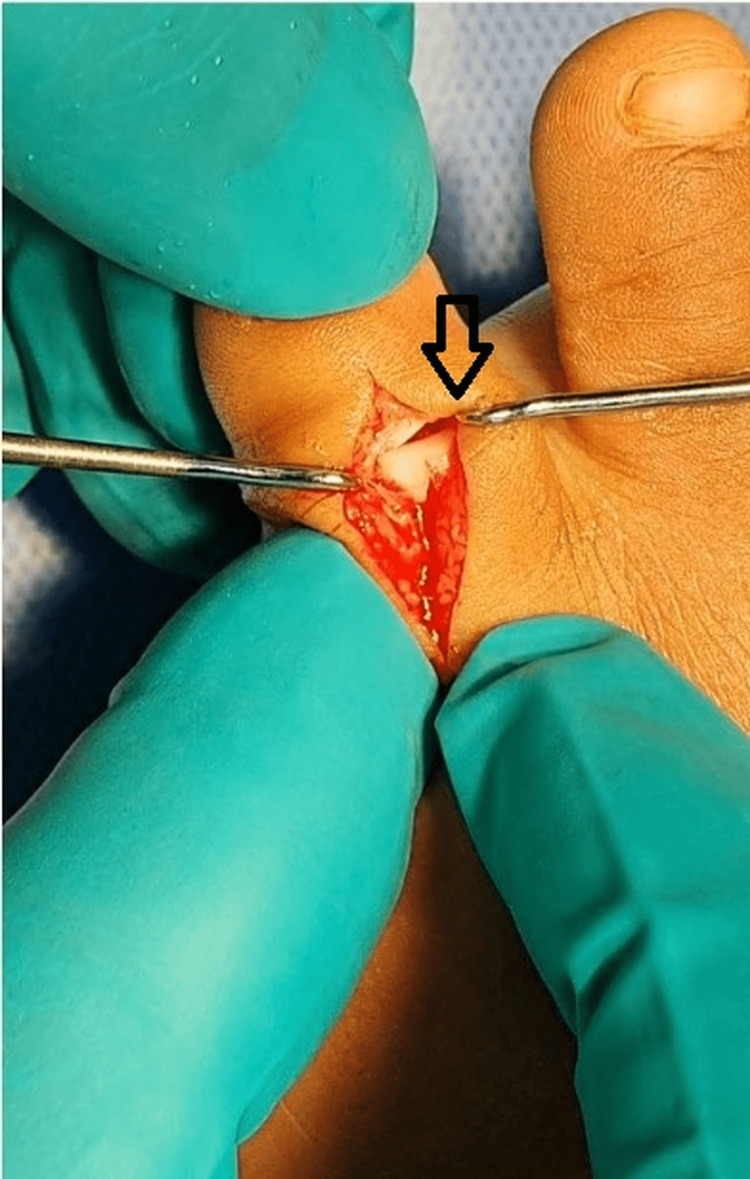
Intraoperative photograph showing the surgical exposure via dorsal midline incision. A freer elevator delineates the avulsed medial collateral ligament (arrow) interposed within the joint space

**Figure 3:**
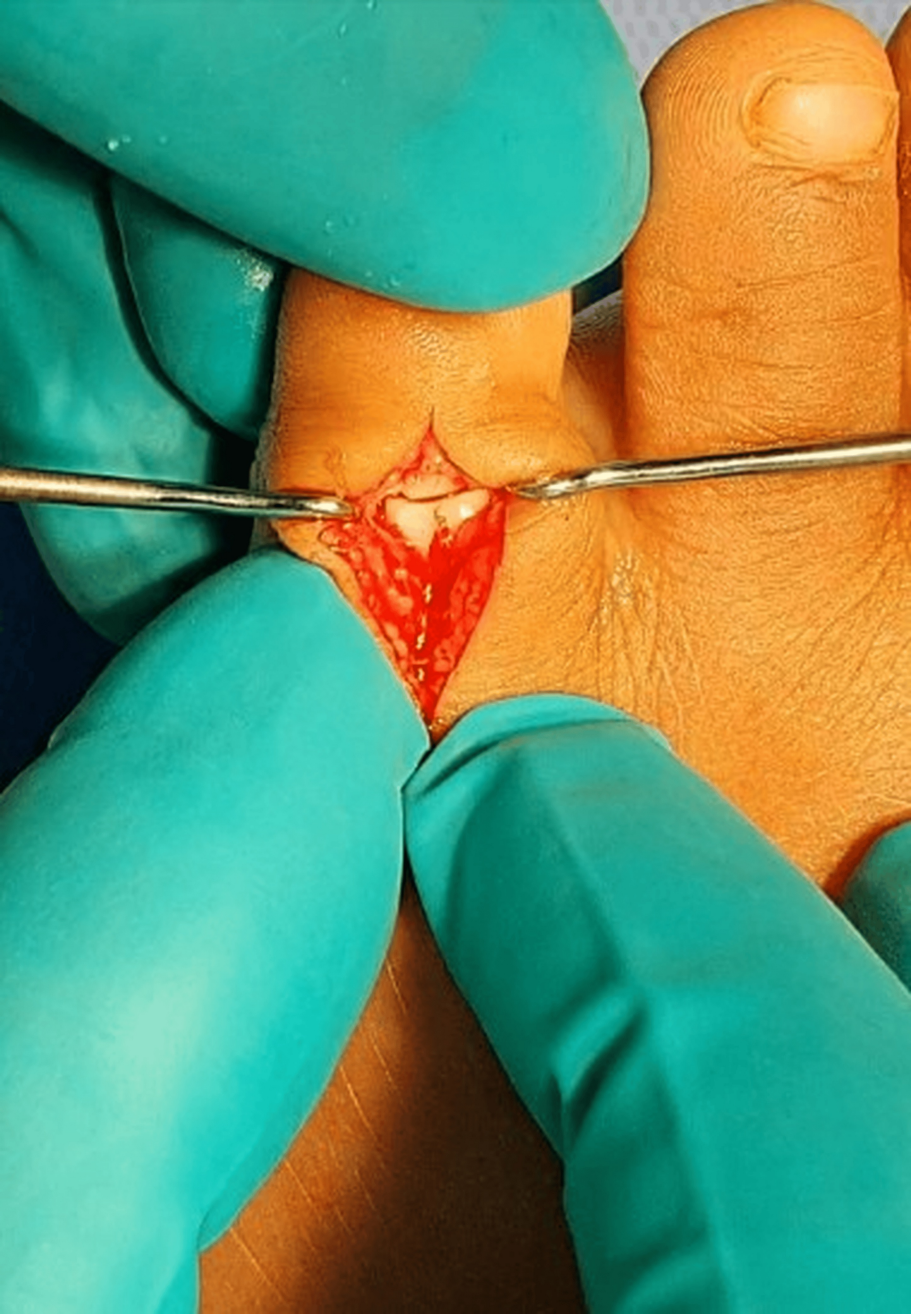
Intraoperative photograph demonstrating the freer elevator removing the avulsed medial collateral ligament from the joint space

Under general anesthesia, a dorsal longitudinal incision was made over the PIP joint. The extensor tendon was split longitudinally, and a capsulotomy was performed. Intraoperatively, the medial collateral ligament was found completely avulsed and interposed within the joint space, acting as a mechanical block to reduction (Figures 2, 3).

The interposed ligament and soft tissue were carefully removed. Articular surfaces showed minimal cartilage injury. Anatomic reduction was achieved; however, the joint remained unstable due to ligamentous disruption.

**Figure 4:**
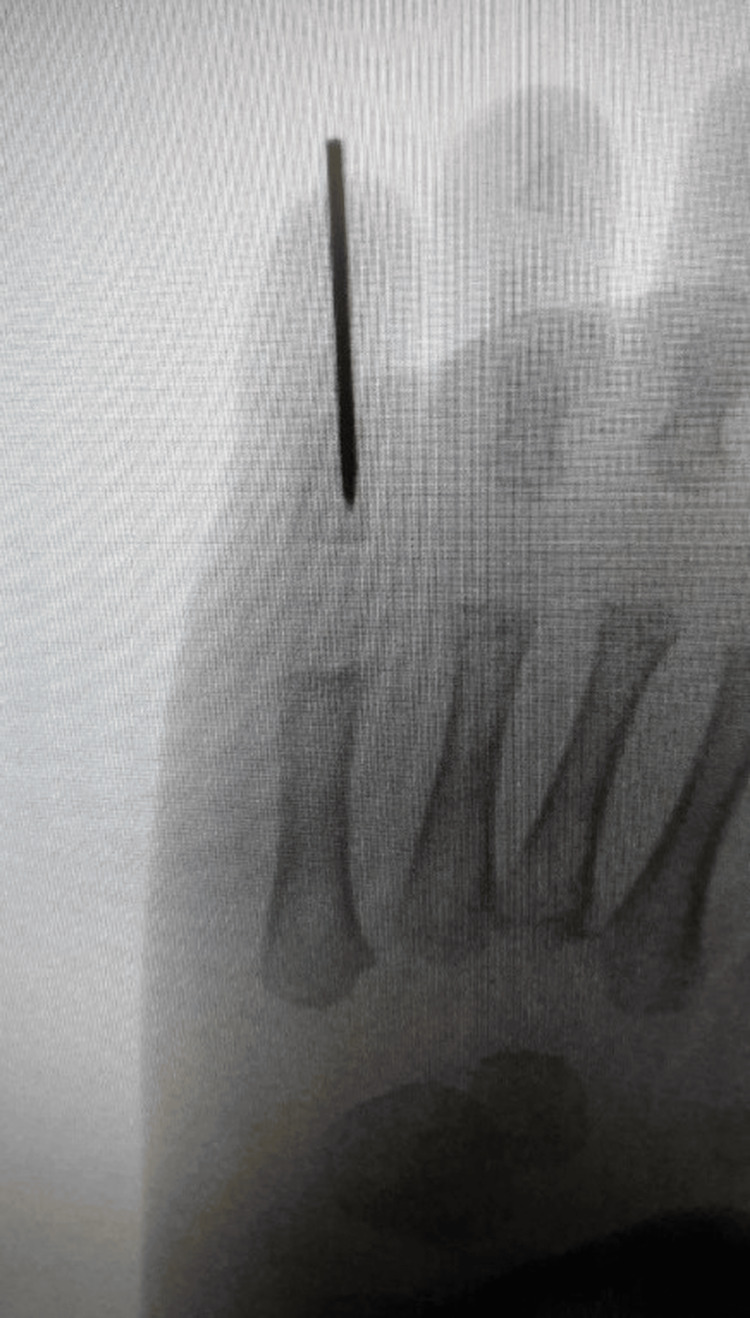
Intraoperative anteroposterior fluoroscopic image confirming satisfactory placement of the 1.6-mm Kirschner wire from the distal phalanx across the reduced PIP joint into the proximal phalanx PIP: proximal interphalangeal

**Figure 5:**
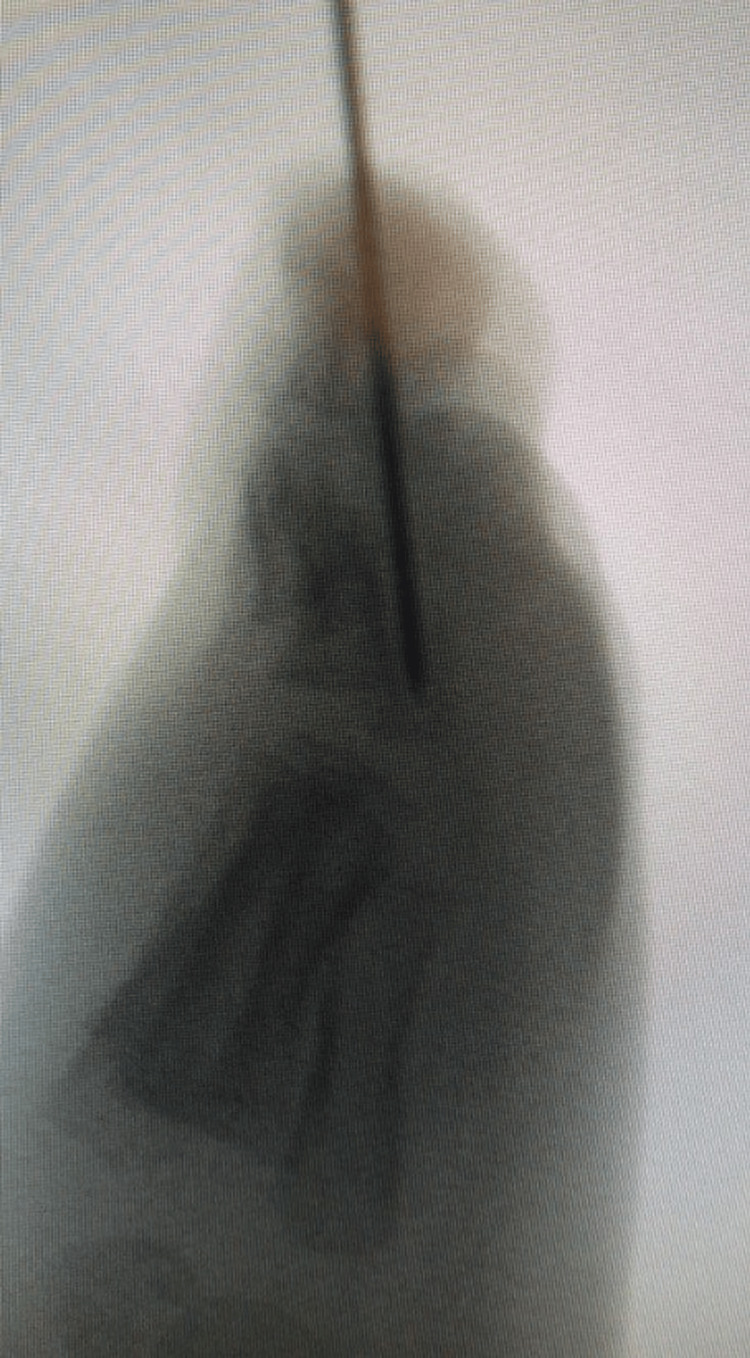
Fluoroscopic view demonstrating the transarticular K-wire in situ with the PIP joint in anatomic alignment in lateral view PIP: proximal interphalangeal

Temporary transarticular fixation was performed using a 1.6-mm Kirschner wire (K-wire) inserted retrograde from the distal phalanx across the PIP joint into the proximal phalanx (Figures 4, 5). Fluoroscopy confirmed satisfactory alignment. The extensor tendon was repaired, and the wound was closed. The toe was buddy-taped to the adjacent fourth toe. The patient tolerated the procedure well. Protected weight-bearing and routine wound care were advised.

At three weeks, the K-wire was removed in the outpatient setting. Radiographs confirmed maintained reduction and early fracture healing. Buddy taping continued for an additional two weeks. At one year, radiographs demonstrated complete fracture healing and maintained joint congruity without malunion, physeal disturbance, or signs of post-traumatic arthritis.

**Figure 6:**
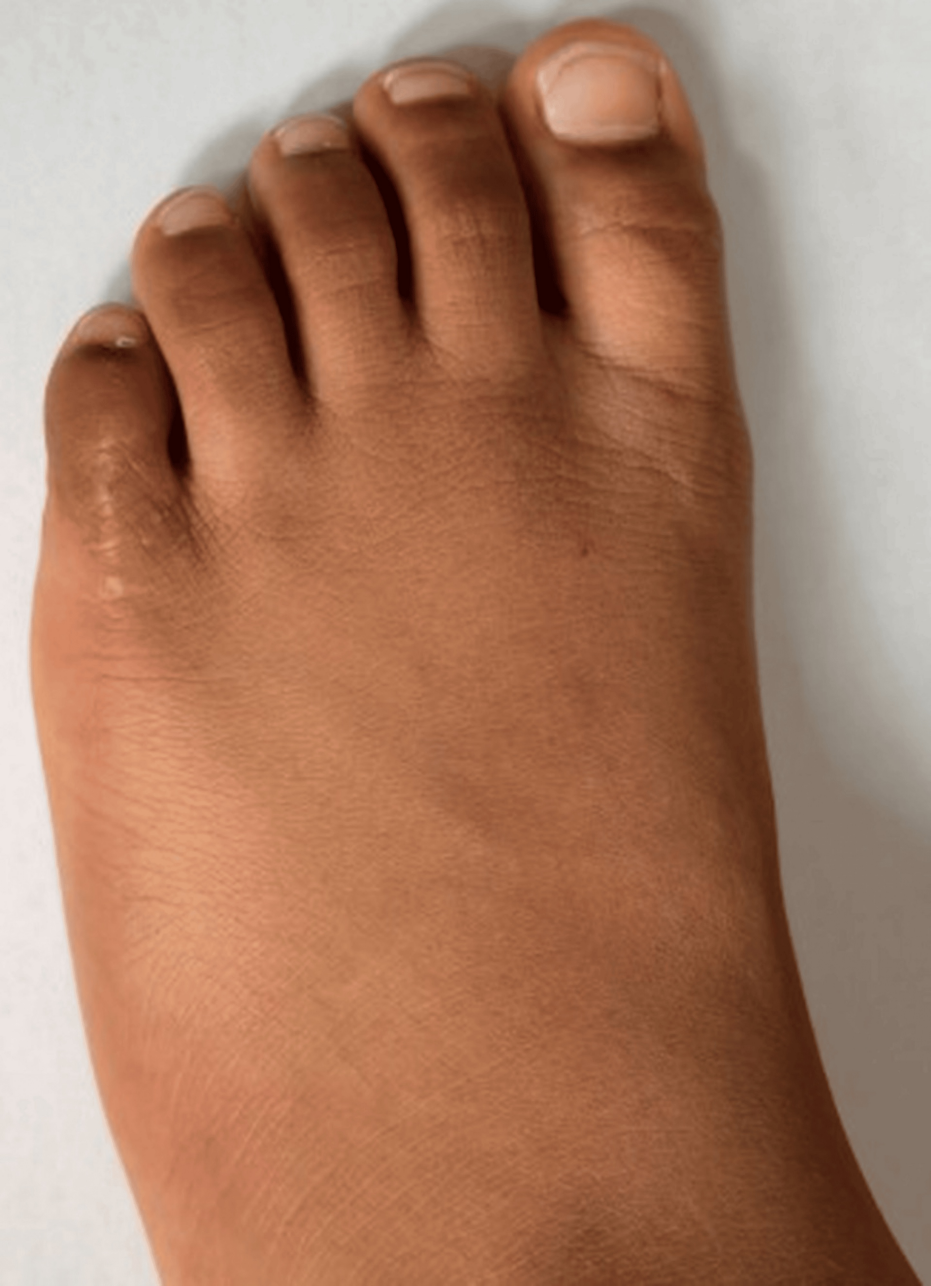
Clinical photograph at six-year follow-up demonstrating excellent cosmetic and functional outcome with no angular deformity, rotational malalignment, or shortening of the fifth toe

At six-year follow-up, clinical evaluation revealed a full range of motion, no angular or rotational deformity, no shortening compared with the contralateral side, and a well-healed scar (Figure 6). The patient was asymptomatic, wore normal footwear, and participated in age-appropriate activities without limitation.

## Discussion

Irreducible PIP joint dislocations of the lesser toes are uncommon in adults and exceedingly rare in children. Yang et al. reported that approximately 38.9% of toe dislocations required open reduction, reflecting the complexity of irreducible cases [1]. In the pediatric population, only isolated reports exist, including cases described by Vij et al., Kushare et al., and Nabi and Kurup [6-8]. Notably, previously published pediatric cases report short-term follow-up. The present case provides one of the longest documented follow-up periods in this age group.

The rarity of these injuries contributes to diagnostic delay. As emphasized in prior reports, a failed closed reduction should raise suspicion for soft-tissue interposition [6]. In young children, clinical assessment may be further complicated by limited history and soft-tissue swelling obscuring deformity.

In our case, complete avulsion and interposition of the medial collateral ligament acted as the mechanical block to reduction. Similar findings have been described by Nabi and Kurup and by Weinstein and Insler, who also identified collateral ligament entrapment as the primary obstructing structure [8,9]. Other reported causes of irreducibility include interposition of the volar plate, joint capsule, plantar plate, or extensor tendon fibers [2,5]. The specific obstructing structure depends on the injury mechanism and joint anatomy. Regardless of the structure involved, recognition of soft-tissue interposition is critical when closed reduction fails.

The dorsal longitudinal approach with extensor tendon splitting provides direct visualization while minimizing neurovascular risk and is consistently recommended in published reports [1,2,6,8]. In our case, this approach allowed identification and removal of the entrapped ligament and restoration of joint congruity.

Temporary transarticular K-wire fixation was used because the joint remained unstable after reduction due to complete ligamentous disruption. Short-term K-wire stabilization (three to four weeks) is widely accepted in similar injuries [2,6,8]. The use of a 1.6-mm wire provided adequate stability for a two-year-old child without risk of iatrogenic fracture.

Direct ligament repair using suture anchors has been described in older children [8]. However, in very young patients with small osseous anatomy, temporary stabilization alone is technically simpler and sufficient, as demonstrated in this case and by Vij et al. [6].

The six-year follow-up is a major strength of this report. Maintenance of alignment, absence of shortening or rotational deformity, and preserved joint motion indicate that neither the injury nor its treatment adversely affected growth. The K-wire did not cross a physis, and its short dwell time likely minimized the risk of growth disturbance.

Published pediatric case reports show good short-term outcomes [6-8], but long-term data beyond one year are lacking. The sustained functional and cosmetic result in this patient supports early anatomic reduction and stable fixation as effective strategies to prevent late deformity or arthritis [1].

This report represents a single case. Radiographs were not obtained at the final follow-up due to parental refusal, and formal functional scoring was not performed. Outcomes were assessed clinically and through patient-reported satisfaction.

## Conclusions

Irreducible PIP joint dislocations of the lesser toes in pediatric patients are rare but require a systematic and timely approach. Failure of closed reduction should prompt early open exploration to identify and remove interposed soft tissue. When instability persists after reduction, temporary K-wire fixation provides reliable stabilization.

This case demonstrates that early surgical intervention can achieve excellent long-term functional and cosmetic outcomes without growth disturbance, even in a very young child. Larger studies and case series are needed to establish evidence-based guidelines for these uncommon pediatric injuries.
